# Our Inheritance

**Published:** 1877-10

**Authors:** 


### OUR INHERITANCE.

Why is it, Doctor, that so much sickness prevails everywhere, demanding the care of so many physicians, and filling our medical institutions with people bearing all manner of diseases?

This is not an unusual question that is propounded the medical man, the answer being readily found in the retrogression of our race. When we stop to consider our inheritance,—the constitutions that are given us by our parents, it ceases to be a wonderment, with the thoughtful, that so many of us are sick or ailing. A lady remarked at the breakfast table, this morning, "It is quite unusual to find a perfectly well person, now-a-days;" to which a gentleman truthfully responded:—That is by reason of the circumstance that "we have no healthful fathers and mothers. This is owing to the dress and habits of our young people—those who are prospectively the fathers and mothers of our race.

When a young woman compresses her lungs within the tight stays that fashion has found to be necessary to the perfect form of body; bares her arms, shoulders and bosom to the night air, after having exercised violently in the ball-room, then partakes of a hearty meal, at an hour when she should have sought rest and sleep in bed; or, as in the ordinary, daily, pursuits of life, she spends all her time with books, music or the entertainment of her company in the parlor, to the exclusion of the more active life that a healthy constitution demands, is it any wonder that she entails upon herself a feeble or sickly constitution, which in turn is imparted to her offspring? Certainly not. Our daughters require to be more thoroughly educated in household duties,—need the wholesome exercise that comes from duty well performed in the kitchen and laundry. When they have learned how to make and bake a loaf of bread, boil a potato; or, in short, how to cook and place upon the table a noon-day meal, and have become accomplished in washing and ironing a shirt, they will have secured a glow to their cheeks and a rotundity of person that bespeak good health, and better fit them for the other accomplishments to be learned in the parlor.

Our boys, too, need reforming. Their principal evil exists in smoking, chewing, drinking and certain secret pollutions of body. The use of tobacco, as it now prevails among our young men, is accomplishing more disastrous results to health, in our opinion, than that of drinking stimulants. And right here is an opportunity for some good and benevolent person, like Francis Murphy, to set in motion another reform, in which young men shall be sought to abstain from smoking, chewing, drinking and secret vices. No young person should ever use tobacco or liquors in any form until he has at least attained his majority, if he would secure to himself a vigorous body.

Until fathers and mothers of the present day find some means of improving the healths of their children, we can not hope for improved health in the generation to come, but rather, that the deterioration shall continue from age to age, until man's allotted time on earth shall be short indeed.

